# Supplementary figures and images for: The NIH BRAIN Initiative’s impacts in systems and computational neuroscience and team-scale research 2014–2023
Source: eLife. 2025 Jul 24;14:RP106136. doi: 10.7554/eLife.106136 (PMC12289304; doi:10.7554/eLife.106136)

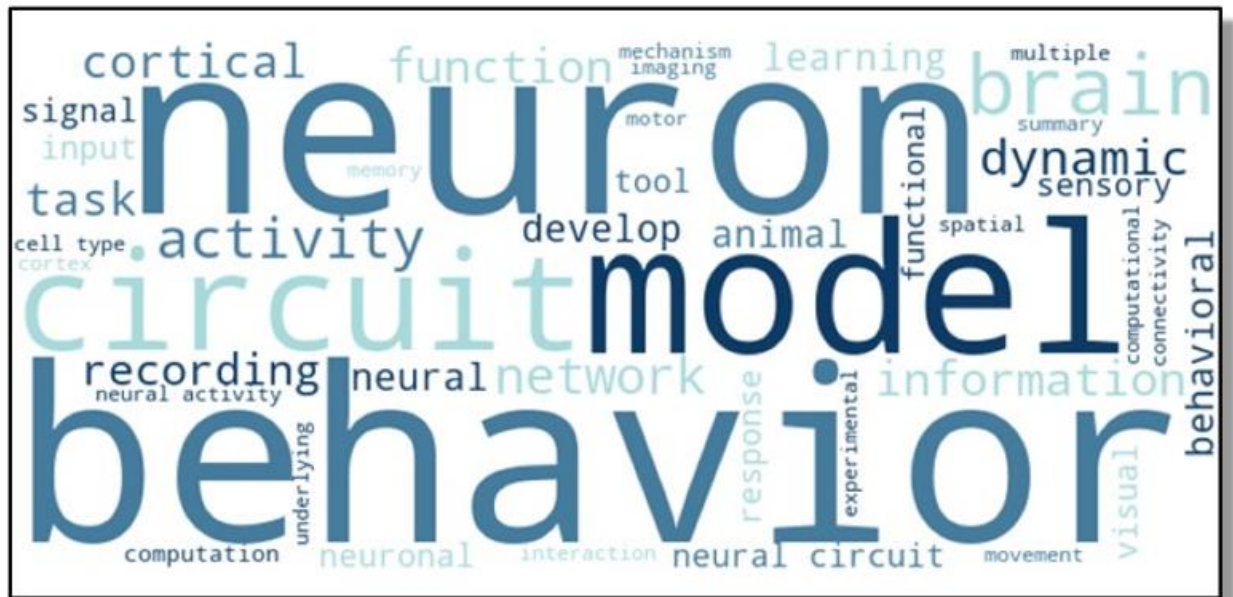

Supplement: Supplementary file 1. — As designed, Behavioral, Systems, and Computational Neuroscience are well represented. [file elife-106136-supp1.pdf]
