# Supplementary material for: The NIH BRAIN Initiative’s impacts in systems and computational neuroscience and team-scale research 2014–2023
Source: eLife. 2025 Jul 24;14:RP106136. doi: 10.7554/eLife.106136 (PMC12289304; doi:10.7554/eLife.106136)
Supplement: Supplementary file 2. — (A) TMM projects with the highest number of cumulative GitHub Stars that produced highly popular computational tools in the form of normative theories, predictive models, and computational algorithms. (B) Highest rated GitHub repositories from TMM awards. These highly popular repositories are supported by multiple projects and programs. Snapshot from November 2024. [file elife-106136-supp2.pdf]

A

Title	T or M or M	GitHub Stars
Next-Generation Calcium Imaging Analysis Methods	Method	1648
Human Neocortical Neurosolver	Model	474
Embedded Ensemble Encoding	Theory	256
Uncovering Population-Level Cellular Relationships to Behavior via Mesoscale Networks	Model	58
Data-driven analysis for neuronal dynamic modeling	Method	56
Methods from Computational Topology and Geometry for Analyzing Neuronal Tree and Graph Data	Method	55
Measuring, Modeling, and Modulating Cross-Frequency Coupling	Model	52
Modeling the structure-function relation in a reconstructed cortical tissue	Model	46
Real-time statistical algorithms for controlling neural dynamics and behavior	Method	33

B

Repository	Purpose	Stars	Watchers
https://github.com/flatironinstitute/CaImAn	Computational toolbox for large-scale Ca+2 imaging analysis	540	50
https://github.com/flatironinstitute/CaImAn-MATLAB	Matlab pipeline for large scale Calcium imaging analysis	236	60
https://github.com/zhoupc/CNMF_E	Constrained Non-negative Matrix Factorization for MicroEndoscopic data	128	53
