# Supplementary material for: The NIH BRAIN Initiative’s impacts in systems and computational neuroscience and team-scale research 2014–2023
Source: eLife. 2025 Jul 24;14:RP106136. doi: 10.7554/eLife.106136 (PMC12289304; doi:10.7554/eLife.106136)
Supplement: Supplementary file 3. — * Manuscripts that attest to early translational impact. [file elife-106136-supp3.pdf]

PMID	Manuscript Title	Altmetric
**TargetBCP R01**		
31473062	A Rare Mutation of β1-Adrenergic Receptor Affects Sleep/Wake Behaviors.	1380
37425937	Neuronal wiring diagram of an adult brain.	1072
36007021	A cognitive process occurring during sleep is revealed by rapid eye movements.	1029
32939091	Deep posteromedial cortical rhythm in dissociation.	801
31619542	Mutant neuropeptide S receptor reduces sleep duration with preserved memory consolidation.	775
31150625	Human Gut Microbiota from Autism Spectrum Disorder Promote Behavioral Symptoms in Mice.*	775
33057202	PIEZO2 in sensory neurons and urothelial cells coordinates urination.	695
31642810	An Integrated Brain-Machine Interface Platform With Thousands of Channels.	628
38092914	A transcriptomic taxonomy of mouse brain-wide spinal projecting neurons.	600
**TeamBCP U19**		
32937591	Non-neuronal expression of SARS-CoV-2 entry genes in the olfactory system suggests mechanisms underlying COVID-19-associated anosmia.*	2777
36603070	A mesothelium divides the subarachnoid space into functional compartments.	2215
37730989	Neural circuitry for maternal oxytocin release induced by infant cries.	2095
35381183	The perception of odor pleasantness is shared across cultures.	1419
28445462	Cell diversity and network dynamics in photosensitive human brain organoids.*	891
34381215	Oxytocin neurons enable social transmission of maternal behavior.	824
32554567	Manipulating synthetic optogenetic odors reveals the coding logic of olfactory perception	784
37286598	Antagonistic circuits mediating infanticide and maternal care in female mice.	723
34930847	De novo mutations in childhood cases of sudden unexplained death that disrupt intracellular Ca2+ regulation.*	721
36889318	Mouse spontaneous behavior reflects individual variation rather than estrous state.	694
36572698	Multimodal monitoring of human cortical organoids implanted in mice reveal functional connection with visual cortex.*	654
34381214	A metabolic function of the hippocampal sharp wave-ripple	612
**ROH U01**		
31019317	Speech synthesis from neural decoding of spoken sentences.	3065
34260835	Neuroprosthesis for Decoding Speech in a Paralyzed Person with Anarthria.	2705
32231340	Machine translation of cortical activity to text with an encoder-decoder framework.	1357
32375031	Replay of Learned Neural Firing Sequences during Rest in Human Motor Cortex.	772
33462446	State-dependent responses to intracranial brain stimulation in a patient with depression.*	645

35511978	The geometry of domain-general performance monitoring in the human medial frontal cortex.	614
29779940	Encoding of Articulatory Kinematic Trajectories in Human Speech Sensorimotor Cortex.	609
**TMM R01**		
29397273	Anxiety Cells in a Hippocampal-Hypothalamic Circuit.	943
