# Supplementary material for: The NIH BRAIN Initiative’s impacts in systems and computational neuroscience and team-scale research 2014–2023
Source: eLife. 2025 Jul 24;14:RP106136. doi: 10.7554/eLife.106136 (PMC12289304; doi:10.7554/eLife.106136)
Supplement: Supplementary file 4. [file elife-106136-supp4.docx]

***What phrase would you use to describe [the BRAIN Circuits Program] science?***

- “Ambitious projects that integrate the interests of multiple BRAIN ICs.”
- “Transformative, multidisciplinary studies of neural circuits and behavior.”
- “Capturing the brain in action.”
- “High-risk high-reward collaborative Systems & Computational Neuroscience”
- “Putting it all together, from biology to behavior.”
- “Linking neural circuit dynamics with behavior.”
- “The Science of Complex Neural and Behavioral Function”

***How has the science stimulated and supported by [the BRAIN Circuits Program] advanced the science of your IC?***

- “[It has stimulated] Team science involving identified cell types and computational modeling.”
- In particular [the BRAIN Circuits Program] endeavors that supported studies to optimize large scale recordings and modulation of the CNS technologies and Targeted Brain Circuits BCP are beginning to pave the way for a more detailed examination of mental health relevant behaviors.”
- “The TargetedBCP program . . . have produced some interesting research that gets at the heart of NINDS mission: ‘to seek fundamental knowledge about the brain and nervous system’.”
- “The NIMH human basic neuroscience portfolio has benefitted from BRAIN investments in ROH [Research Opportunities in Humans]. The number of NIMH applications proposing studies of human neurosurgical patients has increased dramatically over the last 10 years (from near zero to multiple applications each council round).”
- “Many of the [the BRAIN Circuits Program] applications have developed new tools and sensors that are being leveraged in a variety of projects in basic behavior and neuroscience at NIMH that examine neural circuit function in behaving animals.”
- “Three main 'footprints' right now - 1) more integration of computational approaches integrated into behavioral and cognitive neuroscience' projects . . . a big advance over the behavioral pharmacologic approaches still common in NIDA grants. 2) increases in 'multimodal' / multidimensional and higher-resolution measurement of behavior, even in the context of straightforward, task-driven cognitive neuroscience proposals, 3) more consideration of ecologic validity and use of technology to test neurobehavioral processes in naturalistic environments.”

***Briefly describe the science you see as the highest priority areas/approaches to be put forward by [the BRAIN Circuits Program]***

- “The themes cut across IC priorities. If we don't follow up on the last decade of investments in [BRAIN Initiative] portfolios, we lose out on much of the momentum built on both those fronts. The brain does not operate according to IC priorities, and [the BRAIN Circuits] portfolio offers the unique opportunity to not artificially silo the brain and instead study it as a whole.”
- “To provide support to collaborative efforts between theoretical and experimental neuroscientists to leverage the latest advances in the field of AI to generate testable hypotheses about how the brain circuits process incoming information and how at a circuit level the activity of the brain gives rise to behavior . . ..”
- “Computational modeling approaches that DO NOT use AI.”
- “Compared to significant strides made in understanding neural circuits function in relation to complex behaviors, studies examining mechanistic relationship between developing brain circuits and behaviors are lagging . . . Encouraging this type of mechanistic, normative developmental studies would allow researchers to identify how deviations in neurodevelopmental trajectories of neural circuits might be linked to maladaptive behaviors observed across a range of brain disorders.”
- “I am worried about . . . support for systems neuroscience, especially for higher budget, collaborative cutting-edge projects that involve more risk.”
- “Cell-specific neurophysiology techniques . . . need BRAIN support to develop.”
- “Integrated approaches to understanding functional neural circuits with cell-type specificity. This area of research builds on molecular tools that have been developed (in part) by BRAIN and will provide the necessary foundation (and potential targets) for the efforts proposed under the Precision Molecular Circuit Therapies Domain.”
- “Computational models and analytical approaches for understanding high density sampling of neural signals. Technological advances in data acquisition systems . . . have revolutionized neurophysiology. However, there is a need for next generation models and analytics that can interpret neural signals collected simultaneously across multiple brain regions to provide a better understanding of the real-time neural system dynamics that support cognitive, affective, social, and motor behaviors. These approaches, and the knowledge gained from them, will be critical for the success of closed-loop neuromodulatory systems and therapies.”
- “General theoretical frameworks of neural circuit function and behavior that consider the circular relationship between perceptual input and behavioral output in the context of control systems . . ..”
- Further expansion of the BBQS program to include additional measures of sensory perception from the animal's perspective. Development of tools that enable more precise measures of motor output (e.g., flexible electrodes in muscles) that are used alongside continues measures of behavior and neural activity . . ..”
- “Within these goals we prioritize comparative approaches and advancement of computational models that are a synthesis of theory-guided (supervised) and data-driven approaches.
- “We need to continue a [TeamBCP] with greater emphasis on formal/computational modeling and comparative thinking.”
- “[Investigations] providing rationales that link circuit function across evolution and species, even if they are studying only one species in a project.”
- “I see a need for continued, growing support and requirement of use of computational approaches.”
